# Bacteriocins for Safety of Animal-Derived Foods: Systematic Mapping, Multilevel MIC Analysis, Food-Matrix Applications, and Emerging Antiparasitic Evidence

**DOI:** 10.3390/foods15203582

**Published:** 2026-10-09

**Authors:** Rosario De Fazio, Pierluigi Aldo Di Ciccio, Rosangela Odore, Vincenzo Musella, Paola Roncada, Cristian Piras

**Affiliations:** 1 Department of Health Sciences, Magna Græcia University of Catanzaro, 88100 Catanzaro, Italy; rosario.defazio@studenti.unicz.it (R.D.F.); musella@unicz.it (V.M.); roncada@unicz.it (P.R.); 2 Department of Veterinary Sciences, University of Turin, Largo Braccini 2, 10095 Torino, Italy; pierluigialdo.diciccio@unito.it (P.A.D.C.); rosangela.odore@unito.it (R.O.)

**Keywords:** bacteriocins, MIC, multilevel analysis, mixed-effects model, biopreservation, animal-derived foods, foodborne pathogens, food matrix, antiparasitic evidence

## Abstract

Foodborne bacterial hazards remain a major challenge for animal-derived food chains, whereas evidence for antiparasitic effects of bacteriocins is much smaller and mechanistically heterogeneous. This systematic review, reported in accordance with PRISMA 2020, used a comprehensive PubMed, Scopus, and Web of Science search strategy combining pathogen-specific queries with high-sensitivity terms for specific bacteriocin names/classes, pathogen synonyms, strain/pathotype/serovar terms, food applications, parasite synonyms, and known producer-strain names. Across the reconciled evidence base, 577 MIC observations from 146 studies were curated; 312 exact conventional MIC observations from 94 studies formed the primary quantitative tier. The primary inferential analysis used a one-stage linear mixed-effects model of individual log10-transformed MIC observations, with bacteriocin family and broad target category as fixed effects and random intercepts for study, study-strain combination, and bacteriocin identity. Both bacteriocin family (F(11, 52.58) = 3.568, *p* < 0.001) and target category (F(6, 115.42) = 4.219, *p* < 0.001) were significant global terms. Random-intercept variances were 1.098 for study, 0.015 for study-strain, 0.015 for bacteriocin identity, and 0.099 for the residual component. Target-specific study-level aggregations are presented only as descriptive secondary summaries and are not used for inverse-variance inference. The food-application dataset contains 204 unique direct food-matrix studies, 192 involving animal-derived or mixed animal/plant matrices. The dedicated dose-versus-MIC audit included 199 studies; five permitted a direct within-study ratio/equivalence interpretation, whereas most applications were not directly comparable with broth MICs. The antiparasitic mapping identified 11 studies: separately tested anti-Giardia bacteriocin evidence, nine Trichinella studies spanning separately administered enterocins and producer/probiotic strains, and one Cryptosporidium sensitivity-tier preprint. No antiparasitic food-matrix challenge study was identified.

## 1. Introduction

Foodborne diseases and spoilage remain a major public health and food-chain sustainability challenge, with significant impacts in terms of morbidity, socio-economic costs, and food waste [1]. The complexity of food ecosystems (raw materials, processing, distribution, and consumption) facilitates the entry and/or persistence of pathogenic microorganisms throughout the production chain, while the emergence and spread of antimicrobial-resistant strains reduces the effectiveness of traditional control strategies [2]. In this context, interest is growing in biopreservation approaches and mitigation technologies that are effective, selective, and consistent with current demands for safety, naturalness, reduced use of chemical additives, and improved shelf-life. In meat and meat products, it has been observed that lactic acid bacteria and their antimicrobial metabolites, including bacteriocins, can contribute to the inhibition of spoilage and pathogenic bacteria and to shelf-life extension, especially when used in combination with other hurdle technologies. For example, bacteriocin-based treatments have been reported to reduce *Listeria monocytogenes* in ready-to-eat turkey products, while plantaricin produced by *Lactiplantibacillus plantarum* 2C12 showed effectiveness in beef meatballs comparable to nitrite treatment against *Escherichia coli*, with pathogen counts below the detection limit during refrigerated storage and without significant physical or nutritional changes [3]. In dairy products, bacteriocins are also particularly promising because they can inhibit or delay spoilage microorganisms; for example, plantaricin FB-2 produced by *Lactiplantibacillus plantarum* FB-2 has been shown to significantly extend the shelf life of both raw and pasteurized milk at selected concentrations, while also slowing the deterioration of sensory attributes during storage [4].

Bacteriocins are generally peptides produced by bacteria (particularly, though not exclusively, lactic acid bacteria) that can inhibit or kill competing microorganisms through a variety of mechanisms, including membrane permeabilization, interference with cell-wall biosynthesis, and disruption of energy metabolism [5,6]. Many bacteriocins display stability under acidic conditions, tolerance to moderate heat treatments, and synergy with hurdle technologies (e.g., refrigeration, acidification), features that make them attractive for food applications. Some, such as nisin, are already used at industrial level [7]; however, antimicrobial activity is strongly context-dependent, being influenced by the food matrix, processing conditions, physiological state of the target, adaptive responses, and structural barriers (particularly relevant for Gram-negative bacteria) [8,9]. Consequently, an up-to-date and comparable overview of the experimental evidence on bacteriocins that are truly active against food-relevant pathogens is needed.

A further, explicitly secondary component concerns emerging antiparasitic evidence. Direct peptide data and experiments using bacteriocinogenic or probiotic producer strains are therefore not treated as equivalent. The comprehensive search recovered activity of separately tested bacteriocin preparations against *Giardia lamblia* as well as a larger *Trichinella* literature, but these studies are predominantly in vitro, ex vivo, or murine and do not constitute direct evidence of parasite control in food matrices.

In light of these considerations, this review aims to: (i) systematically map bacteriocins reported as active against bacterial targets relevant to food safety while distinguishing pathogen-specific food-safety evidence from broader antimicrobial-spectrum evidence; (ii) extract all eligible conventional MIC observations and perform a one-stage observation-level multilevel analysis that preserves study, bacteriocin and strain structure, with target-specific study-level aggregation only as a descriptive secondary summary; (iii) map direct applications in animal-derived food matrices and classify each intervention as direct bacteriocin addition, producer/protective culture, delivery system, or combined hurdle; and (iv) map emerging antiparasitic evidence using explicit separately tested peptide versus producer-strain categories without equating therapeutic animal-model effects with food biopreservation. Target selection is anchored to European zoonosis surveillance and microbiological food-safety criteria [10,11].

## 2. Methods

This systematic review was conducted and reported in accordance with the PRISMA 2020 statement. No prospective review protocol was registered. The primary quantitative synthesis used a one-stage multilevel mixed-effects analysis of MIC observations. The screening workflow incorporated GPT-6 Sol (OpenAI) solely as a prioritization aid. The automated prioritization step was used exclusively to organize record retrieval and was not used as an independent eligibility criterion. All deduplicated records, irrespective of priority category, underwent manual title/abstract assessment by two reviewers according to the predefined eligibility criteria, and no record was excluded solely on the basis of the model-generated priority assignment. High- and medium-priority records were systematically sought for full-text retrieval. Low-priority records were evaluated using the available title, abstract, indexing record, and other bibliographic metadata; their full texts were assessed when available but were not systematically sought. Thus, prioritization may have influenced the availability of full-text information for low-priority records, but it did not modify the predefined eligibility criteria or permit automatic exclusion. Full-text eligibility assessment and data extraction were performed independently by two reviewers, and any uncertainties or disagreements arising during title/abstract screening or full-text assessment were resolved by consensus. Records were not excluded solely because the abstract did not mention purification or a minimum inhibitory concentration (MIC). When a full text was unavailable, only information explicitly reported in the title, abstract, indexing record, or other accessible bibliographic metadata was considered; no unreported purification step, MIC method, food-matrix experiment, antiparasitic endpoint, or other methodological or outcome information was inferred. Source status was retained in the audit as FULL TEXT REVIEWED, ABSTRACT ONLY, or NOT RETRIEVED. A record labelled NO USEFUL ABSTRACT INFO was not treated as a scientific exclusion in itself; rather, it indicated that the accessible abstract did not contain directly extractable MIC, food-application, or antiparasitic data for the corresponding evidence stream.

### 2.1. Literature Search Strategy and Duplicate Removal

The hazard panel comprised *Staphylococcus aureus*, *Bacillus cereus*, *Escherichia coli*, *Salmonella* spp., *Campylobacter* spp., *Yersinia* spp., *Clostridium botulinum*, *Clostridium perfringens*, *Listeria monocytogenes*, *Giardia* spp., *Anisakis* spp., *Trichinella* spp., *Toxoplasma gondii*, and *Cryptosporidium* spp. The complementary parasite search also included *Taenia* spp. and *Sarcocystis* spp. to assess additional hazards associated with animal-derived foods. Regulation (EC) No 2073/2005 was used as one regulatory reference for microbiological criteria, not as a comprehensive list of foodborne pathogens. Epidemiological relevance was assessed at the most specific level available: STEC/EHEC and serotype/pathotype information for *E. coli*; non-typhoidal *Salmonella enterica* with serovar recorded where reported; *C. jejuni*/*C. coli* for *Campylobacter*; pathogenic *Y. enterocolitica* bioserotype/virulence information where available; and toxigenicity/toxin relevance for *S. aureus*, *B. cereus*, *C. perfringens* and *C. botulinum*. Reference or clinical strains lacking explicit foodborne-hazard characterization were retained as broader antimicrobial-spectrum evidence but were not interpreted as proof of control of the corresponding foodborne toxin or syndrome.

The literature search was conducted in PubMed, Scopus, and Web of Science using two complementary components: pathogen-specific title/abstract queries and a high-sensitivity search including bacteriocin*, nisin, enterocin*, plantaricin*, pediocin*, sakacin*, leucocin*, curvacin*, curvaticin*, lacticin 3147, bavaricin*, carnobacteriocin*, carnocyclin*, subtilosin*, subtilin, microcin*, micrococcin*, lantibiotic*, lanthipeptide*, bacteriocin-producing, and bacteriocinogenic, together with pathogen synonyms, species-level terms, food-matrix terms, parasite synonyms, and a targeted known-producer-strain block. The high-sensitivity exports comprised PubMed (*n* = 9734), Scopus (*n* = 1372) and Web of Science (*n* = 1637) source rows. Complete database-specific search strings are reported in Supplementary Tables S1 and S14.

The core PubMed scheme was (bacteriocin[Title/Abstract]) AND (<pathogen name>[Title/Abstract]). The corresponding Scopus and Web of Science forms were TITLE-ABS-KEY(bacteriocin) AND TITLE-ABS-KEY(<pathogen name>) and TS = (bacteriocin) AND TS = (<pathogen name>), respectively. The original pathogen-specific searches were last run in PubMed, Scopus, and Web of Science on 26 January 2026. The expanded high-sensitivity searches were last run in Scopus on 4 September 2026, and in PubMed and Web of Science on 5 September 2026. The complementary high-sensitivity queries broadened bacteriocin nomenclature, pathogen and parasite synonyms, and food-matrix terminology. No language or publication-date restriction was applied to the high-sensitivity component; document type was assessed during screening rather than used as a database filter.

All citations were exported, reconciled across databases, and deduplicated using DOI, PMID, normalized title and bibliographic metadata, with Mendeley retained as the reference-management layer. Technical duplicate exports were distinguished from record-level bibliographic duplicates. Across all searches, 25,499 source rows were identified; 1325 technical duplicate rows and 11,474 record-level duplicates were removed, yielding 12,700 unique bibliographic records. Within the complementary sensitivity component, 7402 records underwent high-sensitivity screening; 5433 were excluded at first-stage screening and 1969 were retained. These retained records were then reconciled with the core pathogen-specific screening stream; after cross-stream reconciliation and prioritization for MIC, target activity, food-matrix or antiparasitic eligibility, 1591 unique records proceeded to retrieval/eligibility assessment. Retrievable or supplied full texts were assessed in full. For records without an available full text, only explicitly reported abstract/indexing information was extracted. Records were not assigned methods or outcomes that were absent from the accessible source. Retrieval status and scientific eligibility were coded separately, so NOT RETRIEVED did not itself constitute an exclusion reason. The complete arithmetic, access status and exclusion coding are reported in Supplementary Tables S2, S8 and S20.

### 2.2. Bacteriocins Against Bacteria

#### 2.2.1. Preliminary Filtering

Title/abstract screening did not use “purified/purification” or “MIC/minimum inhibitory concentration” as automatic exclusion criteria. These terms were used only as prioritization signals because relevant studies may report purification status or MIC exclusively in the full text, or may refer directly to a named bacteriocin without using the generic term “bacteriocin”. Records were retained when the title/abstract suggested a defined bacteriocin or bacteriocin class, bacteriocinogenic/protective-culture activity, a direct food-matrix intervention, or a relevant pathogen/parasite endpoint. During the subsequent source review, data were extracted at the highest level supported by the available source. When only an abstract was available, a datum was recorded only if it was explicitly stated; otherwise the record was coded NO USEFUL ABSTRACT INFO for that evidence stream. Retrieval status (RETRIEVED, NOT RETRIEVED, or not yet assessed) was maintained separately from evidence classification and exclusion coding.

#### 2.2.2. MIC Evaluation and Statistical Analysis

Data were extracted at the bacteriocin-target-MIC observation level. For each observation, title, DOI, bacteriocin identity, preparation/purity, target species and strain/serovar/pathotype when available, reported MIC, original unit, conversion to mg/mL where directly valid, quantitative evidence tier, and extraction source were recorded. The primary tier comprised exact conventional MIC values for defined bacteriocins in directly convertible mass/volume or molar units with sufficient methodological support to establish that the value represented a conventional MIC of the bacteriocin itself. MIC50/MIC90, censored or range values, arbitrary-unit endpoints, partially characterized preparations, mixtures, formulation-dependent endpoints and non-conventional diffusion-derived values were retained transparently but excluded from the primary quantitative analysis or reserved for sensitivity analysis. Quantitative values available only from an abstract were retained as abstract-sourced sensitivity evidence unless the methodological and preparation details required for primary eligibility were explicitly documented; no missing method or purification information was inferred from the title or abstract.

MIC values were log10-transformed before quantitative analysis. The primary inferential analysis was conducted at the individual-observation level using a one-stage linear mixed-effects model in JASP version 0.98.1 (JASP Team, Amsterdam, The Netherlands; Mixed Models module). The dependent variable was log10 MIC. Bacteriocin family and broad target category were modeled as fixed effects, and random intercepts were specified for Study_ID, Study_Strain, and Bacteriocin identity. The model was fitted by restricted maximum likelihood (REML). Global fixed effects were tested using Type III sums of squares with Satterthwaite degrees of freedom. Adjusted estimated marginal means were visualized for interpretation, but inferential conclusions were based on the global tests and variance components of the final model.

Study-target aggregation was included only as a descriptive secondary analysis. When a study contributed at least two primary MIC observations for a target, the arithmetic mean of log10 MIC and the empirical within-cell dispersion were calculated to summarize and visualize that study-target cell. These observations were not assumed to be interchangeable replicates of a single biological effect, and the resulting dispersion was not interpreted as a sampling variance for inverse-variance weighting. Cells with a single MIC or zero empirical within-cell variance were retained descriptively. Forest-style displays of study-target means and the corresponding study-target summaries are therefore presented only to show the range and between-study pattern of the available evidence; inferential conclusions are based on the observation-level mixed-effects model. A separate cluster-robust variance estimator was not imposed on constructed study-target variances because observation-level sampling variances were not available; dependence was instead modeled directly through the study, study-strain, and bacteriocin random effects. Analysis inputs and descriptive study-target summaries are provided in Supplementary Tables S9 and S21–S23.

#### 2.2.3. Supplementary Mapping of Animal-Derived Food-Matrix Applications

The food-application mapping was derived from the same reconciled evidence base and complementary food-matrix search terms. A study was counted only when the accessible source explicitly documented that a bacteriocin, BLIS/bacteriocin preparation, bacteriocinogenic protective culture, antimicrobial delivery system, or combined hurdle was tested directly in an actual food matrix or food-model system and reported an interpretable application outcome. Animal-derived matrices included meat and meat products, milk/dairy/cheese, fish/seafood, eggs, and mixed systems containing an animal-derived component. The broader food-application audit also retained plant-derived and unclassifiable food studies for completeness of the reconciled evidence base; analyses described as animal-derived or mixed were restricted to studies containing an animal-derived component. Each eligible application was classified as: (i) direct bacteriocin/BLIS addition; (ii) producer/protective culture; (iii) delivery system/active packaging or coating; or (iv) combined-hurdle/multi-component treatment. Broth-only tests, potential-use statements, isolation from food without application, food-contact-surface studies without a food matrix, and reviews were excluded. When classification was based on an abstract rather than a full text, only matrix, intervention and outcome details explicitly stated in the abstract were entered, and the source was flagged accordingly. Article-level matrix, target, intervention, DOI/source link and outcome are reported in Supplementary Table S5.

#### 2.2.4. Methodological Quality and Reporting Appraisal

Because no validated risk-of-bias instrument is tailored to heterogeneous in vitro bacteriocin MIC studies, an adapted predefined nine-domain reporting/methodological appraisal was applied to the 94 studies contributing primary quantitative MIC observations. Domains were bacteriocin identity, preparation/purity, target strain/serovar/pathotype characterization, exact/convertible MIC endpoint, MIC assay reporting, growth medium, inoculum, incubation conditions, and source verification. Missing information was coded as unclear rather than assumed absent. The instrument is therefore interpreted as a quality/reporting appraisal rather than a clinical risk-of-bias tool. Study-level results are provided in Supplementary Table S4 and are used to qualify quantitative interpretation rather than to automatically exclude studies.

#### 2.2.5. Food Dose Versus MIC Comparability

Food-application doses were compared with MIC values only when bacteriocin identity, target, concentration basis, matrix exposure and units permitted a meaningful numerical comparison. Producer cultures, AU/BU-based activities, surface loadings, encapsulated formulations, films/coatings and multi-hurdle systems were not numerically equated with broth MICs. Each application was therefore classified as directly comparable, not directly comparable, or lacking a matched MIC. The complete audit and explicit reason for every classification are reported in Supplementary Table S6.

### 2.3. Bacteriocins Against Parasites

The dataset was screened for the parasite targets using parasite synonyms (including *Giardia lamblia*, *G. intestinalis* and *G. duodenalis*), individual bacteriocin names/classes, *Taenia* spp., *Sarcocystis* spp., and known producer-strain names so that studies testing bacteriocinogenic strains without naming the peptide in the title/abstract could be recovered. Evidence was classified prospectively for interpretation into: (i) direct peptide-exposure evidence, where an isolated or otherwise separately tested bacteriocin preparation was administered to the parasite/model; (ii) bacteriocinogenic/producer-strain evidence, where a strain explicitly documented as bacteriocin-producing was administered; (iii) indirect probiotic-strain evidence, where bacteriocin involvement was not demonstrated in the current experiment; and (iv) sensitivity-tier postbiotic/preprint evidence. Direct peptide exposure was not assumed to imply direct parasiticidal activity unless parasite killing or viability was measured. These categories were not pooled as equivalent. For abstract-only records, antiparasitic evidence was entered only when the abstract explicitly reported a relevant parasite endpoint; no bacteriocin-mediated effect was inferred from probiotic identity or producer status alone. Murine therapeutic or immunomodulatory studies were explicitly separated from food-matrix biopreservation, and possible repeated use of the same producer strains or research programme was recorded to avoid assuming statistical independence.

## 3. Results

### 3.1. PRISMA Flow Diagram

**Figure 1:**
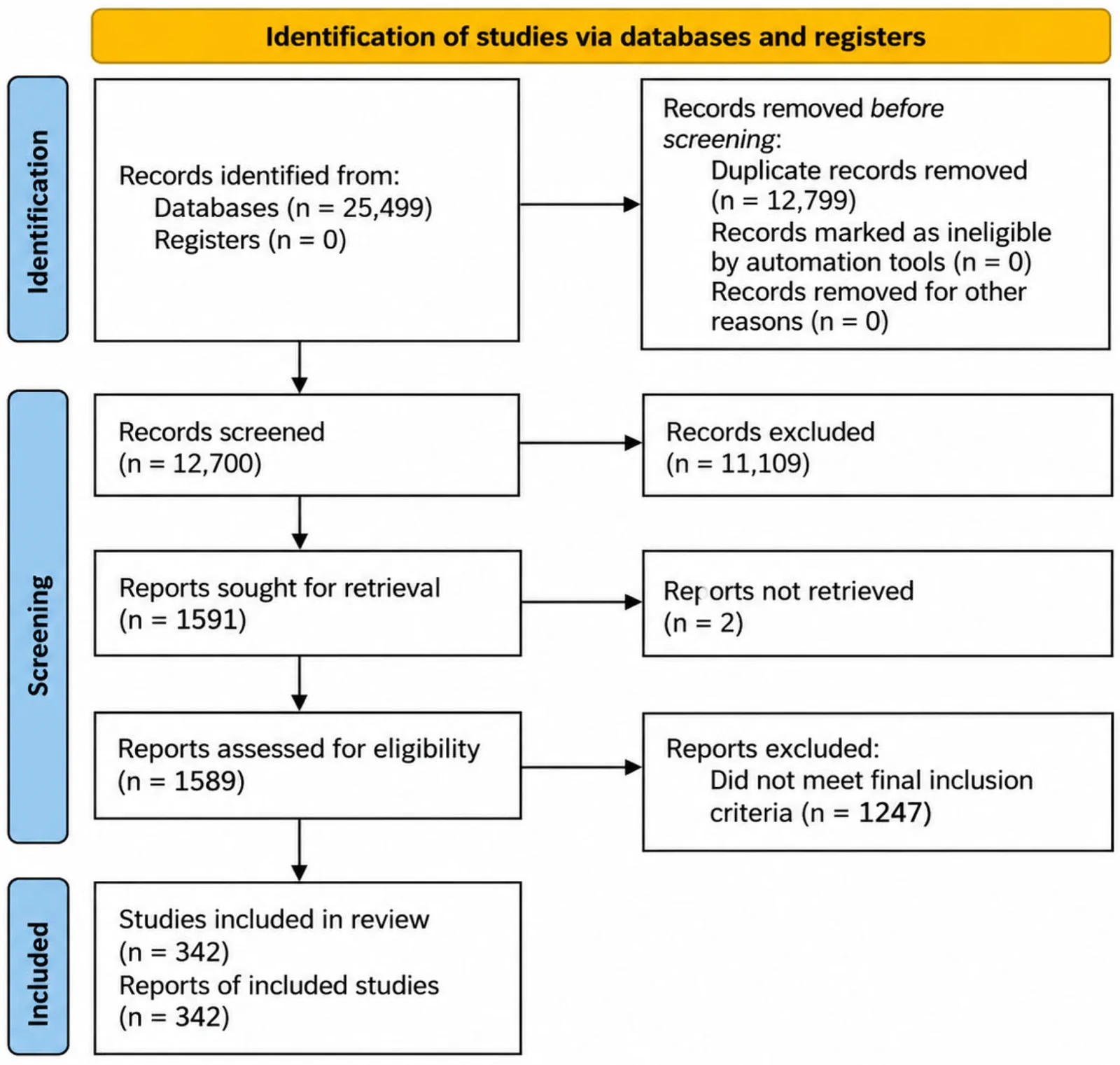
PRISMA flow diagram of study identification, screening, eligibility and inclusion.

The database searches identified 25,499 source rows in total: 12,756 from the core pathogen-specific searches and 12,743 from the complementary high-sensitivity database exports (PubMed 9734; Scopus 1372; Web of Science 1637). Removal of 1325 technical duplicate export rows and 11,474 record-level bibliographic duplicates yielded 12,700 unique records. Within the complementary component, 7402 records entered high-sensitivity screening; 5433 were excluded at first-stage screening and 1969 were retained. These records were reconciled with the core pathogen-specific screening stream, and 1591 unique records proceeded to retrieval/eligibility assessment. Retrievable or supplied full texts were assessed in full. Records remaining supported only by abstract/indexing information were retained as low-prioritization records in the audit and were not assigned unreported methods or outcomes. The curated evidence sets comprise 146 MIC studies (577 MIC observations), 204 direct food-matrix studies, and 11 antiparasitic studies, representing 342 unique publications after accounting for 19 MIC-food overlaps; no additional overlaps involving the antiparasitic set were present in the reconciled publication count. The PRISMA 2020 flow diagram is shown in Figure 1, with detailed search, deduplication and screening data in Supplementary Tables S2, S8 and S20.

### 3.2. Antibacterial MIC Evidence

*Staphylococcus aureus* was represented by 81 primary MIC observations from 52 studies and 59 distinct bacteriocins; *Listeria monocytogenes* by 124 observations from 34 studies and 50 bacteriocins; *Escherichia coli* by 35 observations from 29 studies and 29 bacteriocins; and *Salmonella* spp. by 19 observations from 18 studies and 18 bacteriocins. *Clostridium perfringens* and *Bacillus cereus* contributed 4 and 19 primary observations, respectively. After full-text verification of assay methodology, no *Campylobacter* spp., *Yersinia* spp., or *Clostridium botulinum* observation remained eligible for the exact conventional primary tier; non-conventional agar/spot endpoints were retained in the curated sensitivity evidence instead. Thirty primary observations across seven studies involved targets outside the predefined hazard panel. The counts are explicitly termed bacteriocin-target evidence rather than ‘number of active bacteriocins’, because the same bacteriocin may occur across several targets.

The target categories do not imply equivalent foodborne relevance of every tested strain. Clinical MRSA and laboratory reference strains, for example, contribute to antimicrobial-spectrum evidence but are not interpreted as direct evidence of enterotoxin control in food. Likewise, MICs against *B. cereus*, *C. perfringens* or other toxigenic species demonstrate growth inhibition of the tested strain, not inhibition of toxin synthesis unless the study measured toxin production. Strain, serovar or pathotype information is therefore retained wherever available in Supplementary Table S3 and considered in interpretation.

#### 3.2.1. Evidence Coverage by Target and Bacteriocin Family

**Figure 2:**
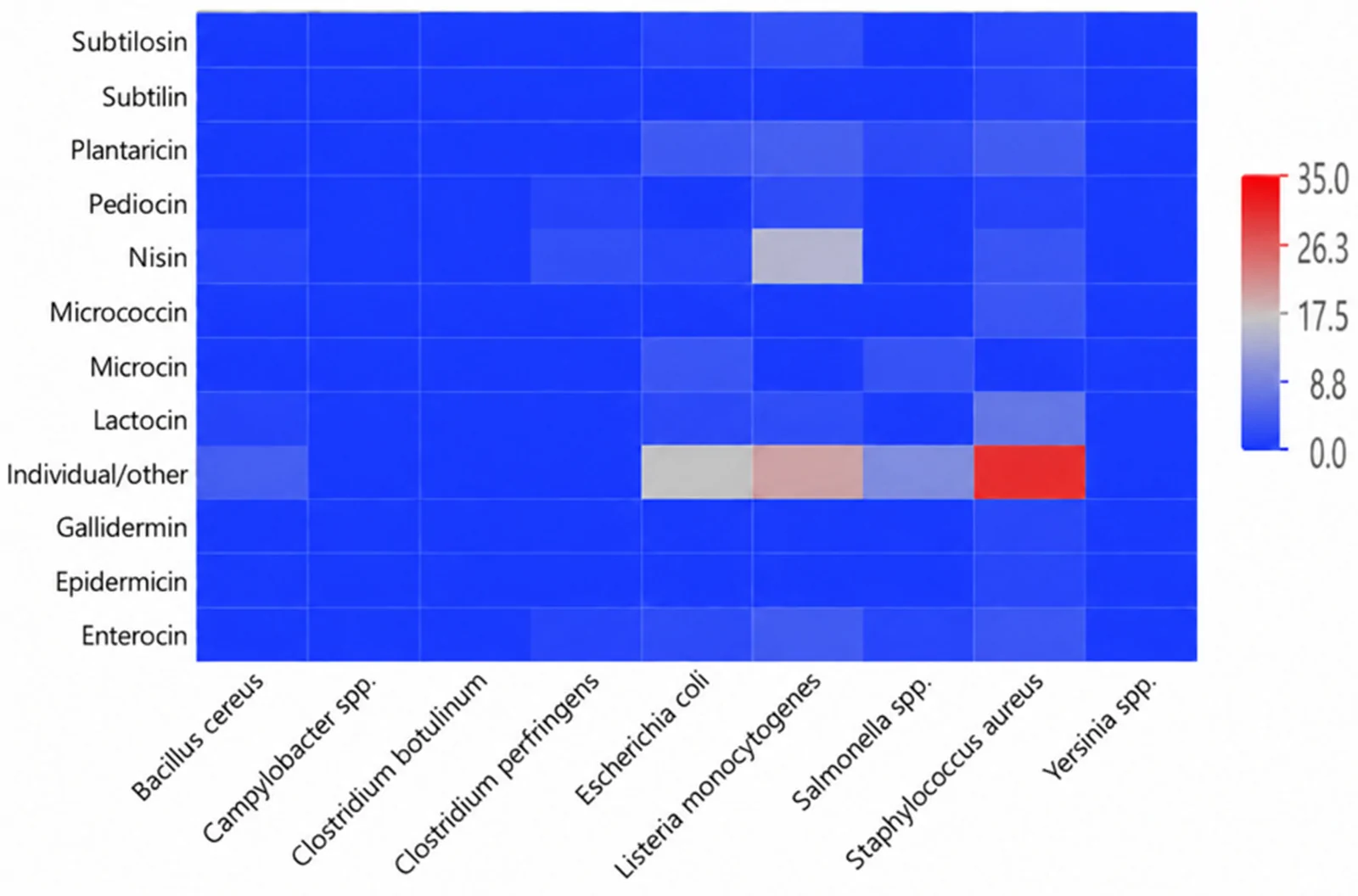
Bacteriocin family × target-pathogen coverage in the primary exact-MIC tier. Cell values are numbers of distinct bacteriocins represented; rare individual families are grouped as Individual/other.

Table 1 summarizes target-level coverage in the primary exact-MIC tier, including the number of MIC observations, distinct bacteriocins, and contributing studies for each predefined target group. Figure 2 provides the complementary bacteriocin-family × target-pathogen heatmap, showing the breadth and density of evidence across bacteriocin families and microbial targets and highlighting areas with limited or absent coverage.

#### 3.2.2. Descriptive MIC Distribution and Study-Target Summaries

To provide a descriptive overview of between-study variability, target-specific study-level summaries were included as a secondary descriptive analysis and are reported in Table 2. These summaries include only study–target cells for which a descriptive mean could be calculated and are presented as unweighted mean log10 MIC values, corresponding back-transformed concentrations, and observed ranges across study–target cells. *Staphylococcus aureus*, *Escherichia coli*, and *Listeria monocytogenes* were represented by 9, 3, and 6 study–target cells, respectively. The broad ranges observed within each target indicate substantial variability among studies, which may reflect differences in bacteriocin identity, strain susceptibility, preparation characteristics, assay conditions, and other experimental factors. Importantly, these values are intended solely to describe the distribution of study-level evidence: they are not treated as pooled effect sizes, and their within-cell dispersion is not used as a sampling variance for inverse-variance inference.

**Figure 3:**
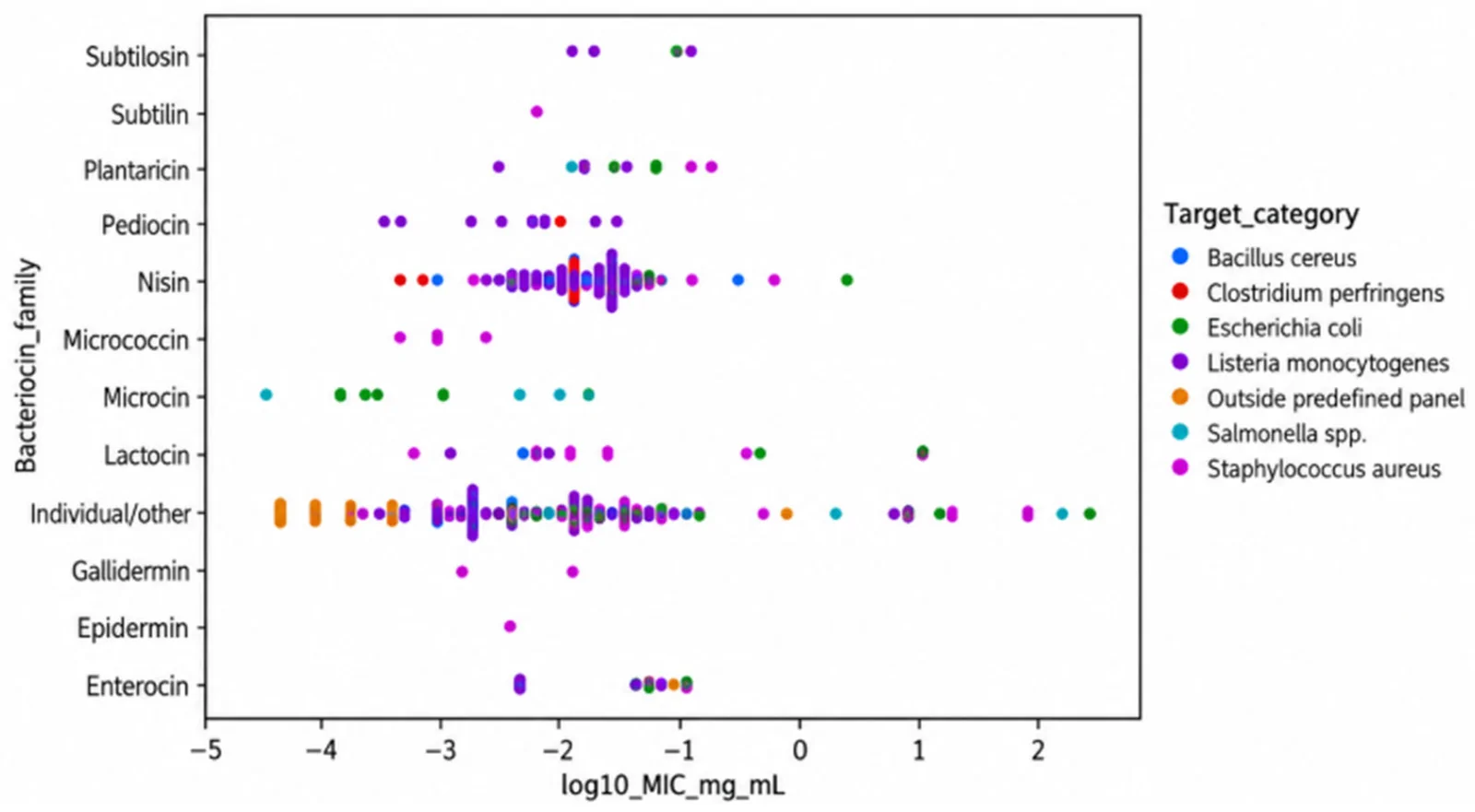
Descriptive distribution of primary minimum inhibitory concentration (MIC) values across bacteriocin families. Each point represents one primary quantitative MIC observation expressed as log10 MIC (mg/mL), with point color indicating the target microorganism. Bacteriocin families are displayed separately to show within-family dispersion and overlap across targets. Lower values correspond to lower observed MICs. The figure is descriptive and should not be interpreted as an adjusted ranking of bacteriocin potency.

A complementary descriptive perspective is provided by the distribution of all 312 individual primary MIC observations shown in Figure 3. In contrast to the study-level summaries in Table 2, this visualization preserves the individual observation structure and illustrates the full range of MIC values across bacteriocin families and microbial targets. Considerable within-family dispersion and extensive overlap among target microorganisms are evident. Several bacteriocin families include very low MIC observations, indicating potentially strong activity in specific bacteriocin–target combinations, but no family forms a clearly separated distribution across the complete evidence base. The visual pattern is also influenced by the markedly unequal representation of bacteriocin families and targets, particularly for sparsely investigated families, while the heterogeneous Individual/other category spans a large proportion of the observed MIC range. Figure 3 should therefore be interpreted as a descriptive representation of the breadth, variability, and structure of the available evidence rather than as an adjusted ranking of bacteriocin potency. Formal inference regarding bacteriocin family and target category is presented separately in the subsequent multilevel mixed-effects analysis.

#### 3.2.3. Multilevel Mixed-Effects Analysis

Of 577 curated MIC observations, 312 exact conventional values in directly convertible units formed the primary quantitative tier and were retained at the individual-observation level. The final linear mixed-effects model included all 312 observations nested within 212 study-strain clusters from 94 studies and 117 bacteriocin identities. The model was fitted by restricted maximum likelihood (REML) and had a deviance of 495.3, log likelihood of −247.6, AIC of 539.3, and BIC of 621.6. Type III tests with Satterthwaite degrees of freedom showed a significant global effect of bacteriocin family on log10 MIC (F(11, 52.58) = 3.568, *p* < 0.001) and a significant global effect of target category (F(6, 115.42) = 4.219, *p* < 0.001). For the random-effects structure, the Study_ID intercept had SD = 1.048 and variance = 1.098, the Study_Strain intercept had SD = 0.124 and variance = 0.015, the Bacteriocin intercept had SD = 0.123 and variance = 0.015, and the residual SD was 0.314 with variance = 0.099. Thus, the dominant variance component was at the study level, while additional study-strain and bacteriocin components were smaller but non-zero. The inferential results therefore indicate systematic differences among bacteriocin families and target categories after explicitly accounting for clustered observations and recurrent bacteriocin identity, without implying a simple potency ranking among individual bacteriocins. The results of the final observation-level mixed-effects model are summarized in Table 3.

**Figure 4:**
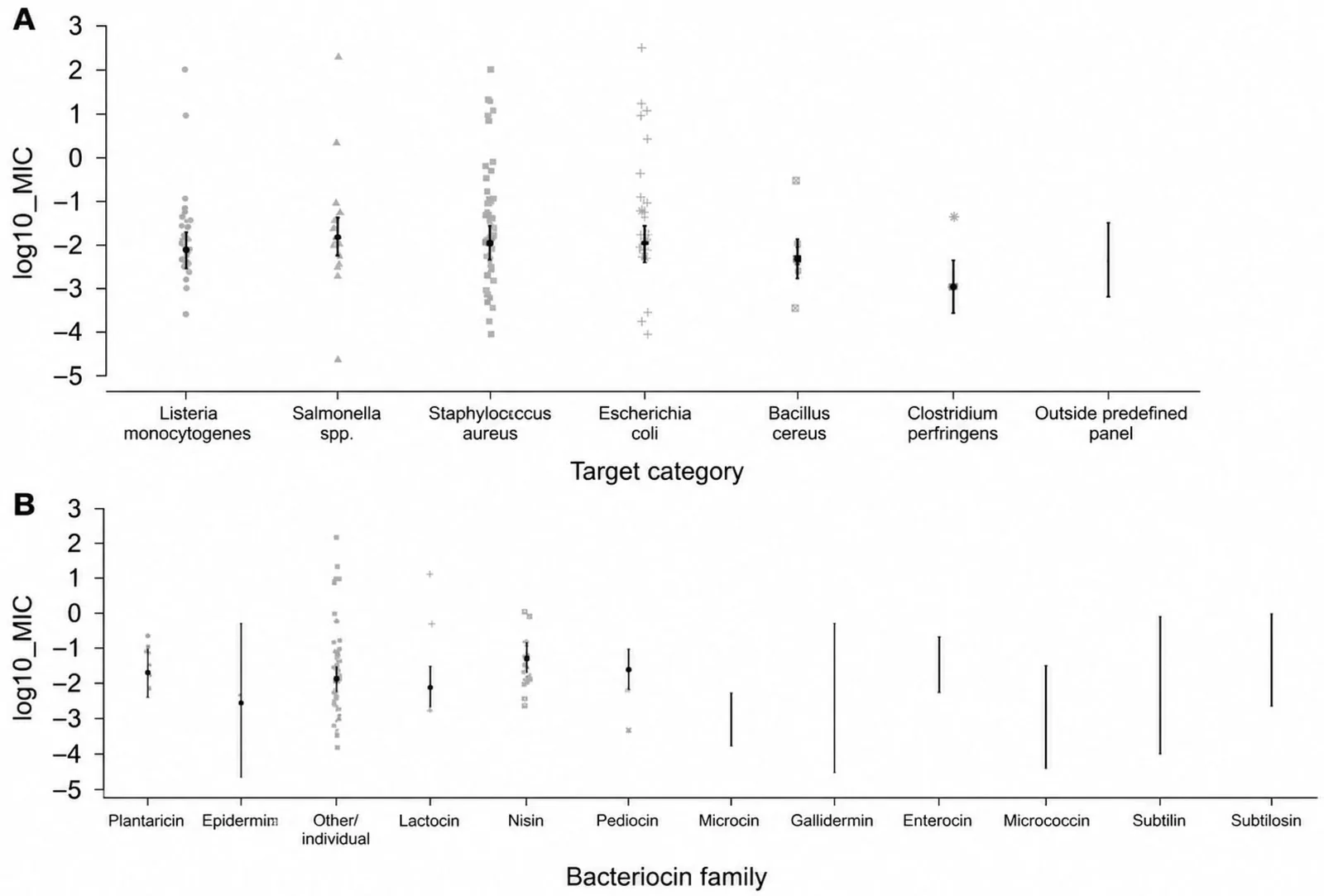
Model-based adjusted estimated marginal means of log10 MIC (mg/mL) with 95% confidence intervals from the final observation-level linear mixed-effects model. Panel (**A**) shows estimates by target category, whereas Panel (**B**) shows estimates by bacteriocin family. Grey symbols represent the individual observed log10-transformed MIC values included in the primary analysis; marker shapes distinguish target categories. Black points indicate the model-based adjusted estimated marginal means (EMMs), and vertical black error bars indicate the corresponding 95% confidence intervals. Estimates are adjusted for the other fixed effect and for the random-intercept structure of Study_ID, Study_Strain, and Bacteriocin identity. Lower values correspond to lower adjusted MICs. The broad confidence intervals for several underrepresented bacteriocin families reflect limited precision rather than absence of activity.

Model-based adjusted estimated marginal means are visualized in Figure 4. Panel A summarizes target-category estimates from the final model, whereas Panel B shows the corresponding family-level estimates. The target-category plot indicates comparatively lower adjusted MIC values for *Clostridium perfringens* and *Bacillus cereus*, intermediate values for Listeria monocytogenes and *Escherichia coli*, and comparatively higher adjusted values for *Salmonella* spp. and *Staphylococcus aureus*. The family panel confirms heterogeneity across bacteriocin families but also shows that several sparsely represented families have broad confidence intervals, so the family effect should be interpreted as a global pattern rather than as a stable rank ordering of all individual families. Target-specific study-level aggregation is presented only for descriptive context and is summarized separately in Table 2.

### 3.3. Methodological Quality and Reporting Appraisal

The adapted nine-domain appraisal was applied to the 94 studies contributing primary exact/convertible MIC data. Fifty-two studies were classified as high/unclear concern, 23 as some concern, and 19 as low concern under the reporting-based algorithm. Bacteriocin identity was defined in 93/94 studies, preparation/purity information was available in 80/94, and target strain/serovar/pathotype characterization was complete in 47/94. The appraisal identifies uncertainty in reporting and methodology and is used to temper interpretation, not to assert poor experimental conduct or to exclude studies automatically (Supplementary Table S4).

### 3.4. Bacteriocin Applications in Animal-Derived Food Matrices

The reconstructed application mapping contains 204 unique studies with direct food-matrix testing. Of these, 166 involved animal-derived matrices and 26 involved mixed animal/plant systems, yielding 192 studies with an animal-derived component; eight were plant-derived and four could not be classified confidently from the available record. The 12 studies without a confirmed animal-derived component were retained only in the broader food-application audit and were excluded from counts and interpretations explicitly described as animal-derived or mixed. Among the 192 animal-derived or mixed studies, 74 used direct bacteriocin/BLIS addition, 67 combined-hurdle or multi-component treatments, 33 producer/protective cultures, and 18 delivery systems such as active films, coatings or packaging. These categories are not interpreted as equivalent evidence of independent bacteriocin efficacy. The full article-level mapping, DOI/source links, matrix, target and intervention type is provided in Supplementary Table S5.

**Figure 5:**
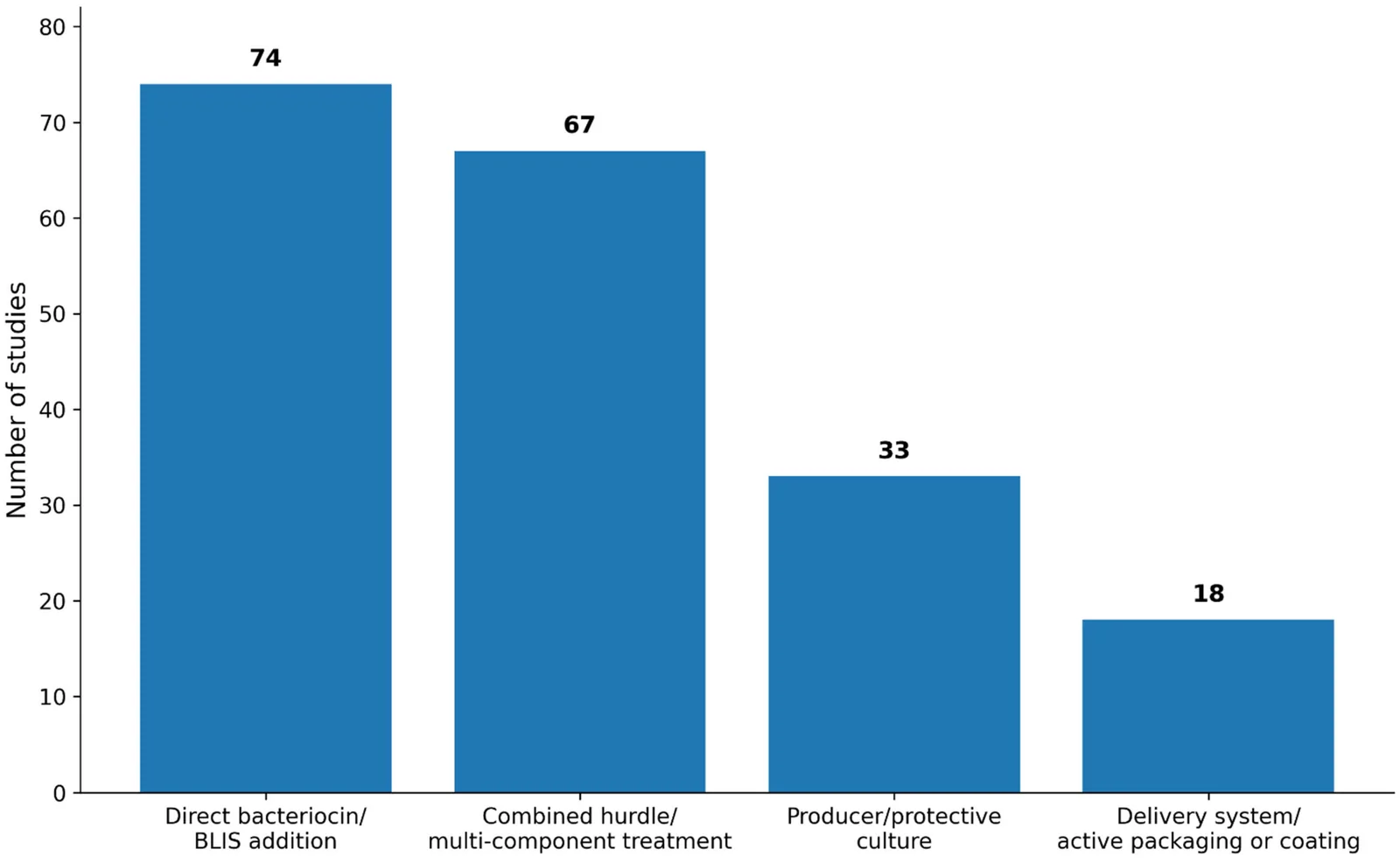
Distribution of direct animal-derived or mixed food-matrix applications by intervention type (*n* = 192 unique studies).

Table 4 and Figure 5 show that direct addition and combined-hurdle studies account for the largest shares of the animal-derived/mixed evidence, while protective cultures and delivery systems form smaller categories. The dedicated dose-versus-MIC audit comprised 199 food-matrix studies. Five allowed a direct within-study ratio/equivalence interpretation: one study reported food doses explicitly as multiples of the corresponding MIC, two supported compatible mass/volume comparisons, one supported an activity-unit ratio within the same study, and one allowed component-wise ratios across mixed units. Two additional records allowed only methodologically limited comparisons, 189 were not directly comparable, and three lacked a matched MIC in the curated dataset. Accordingly, food-application efficacy is interpreted primarily through challenge-test outcomes rather than by assuming that an applied concentration is a simple multiple of the in vitro MIC (Supplementary Table S6).

### 3.5. Bacteriocins Against Parasites

The antiparasitic search identified 11 publications across four evidence categories. Amer et al. [12] tested bacteriocins derived from *L. acidophilus* P106 and *L. plantarum* P164 as separately administered peptide preparations against *Giardia lamblia*. At 50 μg/mL, the P106 bacteriocin reduced mean trophozoite density by 58.3 ± 4.04% in vitro; after five daily oral doses of 50 μg/mouse, intestinal parasite counts were reduced by 81.63%. The P164 bacteriocin was weaker (29.06 ± 3.5% in vitro at 50 μg/mL and 31.38% after the five-dose murine regimen). Nine publications addressed *Trichinella spiralis*, and one 2025 *Cryptosporidium parvum* preprint was retained only as sensitivity-tier postbiotic evidence because the bacteriocin contribution could not be separated from other cell-free-supernatant components [13]. No eligible direct peptide-exposure evidence was identified for *Toxoplasma gondii*, *Anisakis* spp., *Taenia* spp., *Sarcocystis* spp., or the other parasite targets screened.

The *Trichinella* literature was separated into separately administered bacteriocin/enterocin interventions and producer/probiotic-strain interventions. The distinction materially changes interpretation. In the 2022 CECT7121 study, the live *Enterococcus faecalis* producer reduced larval viability and murine parasite burden, whereas isolated AP7121 showed no larvicidal effect at the tested concentrations [14]. Conversely, enterocin M and a durancin-like preparation were administered separately from their producer enterococci in the 2023–2024 Slovak studies and produced measurable immunomodulatory and antiparasitic effects [15,16]. Earlier studies using *E. faecium* CCM8558, *E. durans* ED26E/7 and *L. plantarum* 17L/1 provide producer-strain evidence [17,18,19,20], while the 2015 *L. plantarum* P164 experiment and the 2025 *L. brevis* PQ214320/*B. subtilis* PQ198038 study remain indirect/sensitivity evidence because the current experiments did not isolate a bacteriocin as the causal antiparasitic agent [21,22].

None of the 11 antiparasitic publications tested control of a parasite directly in an animal-derived food matrix. The evidence therefore supports only preliminary mechanistic or therapeutic hypotheses. Table 5 reports study-by-study evidence category, model and key outcome so that effects observed after separately administered peptide exposure cannot be conflated with demonstrated direct parasiticidal activity or with effects of a live producer strain or mixed postbiotic preparation.

Potential overlap within the Slovak research programme was also considered. The 2016 and 2018 studies used related strain panels but differ in sample size and documented experimental protocols and are treated as separate cohorts; the 2022 lymphocyte study uses the same core strains and infection dose and is retained as mechanistic evidence but is not quantitatively pooled. Because no antiparasitic meta-analysis is performed, repeated use of strains or related experimental programmes does not create pseudo-replication in the quantitative synthesis.

## 4. Discussion

### 4.1. Antibacterial Activity

The primary quantitative inference was based on the one-stage mixed-effects model of all 312 exact conventional MIC observations rather than on inverse-variance pooling of aggregated study-target cells. In the final specification, dependence was modeled through random intercepts for study, study-strain combination, and bacteriocin identity. After this adjustment, both bacteriocin family and target category remained significant global terms (Table 3). The study-level variance (1.098) was substantially larger than the study-strain variance (0.015), bacteriocin variance (0.015), and residual variance (0.099), indicating that study-level heterogeneity represents an important source of variation in observed MIC values. The adjusted estimated-marginal-means plots in Figure 4 visualize the corresponding model-based differences and uncertainty. These findings indicate that log10 MIC differs systematically across bacteriocin families and microbial target categories, but they should not be interpreted as a ranking of individual bacteriocins. The target-specific study-level summaries in Table 2 and the observation-level distribution in Figure 3 are presented as descriptive components that illustrate the breadth, dispersion, and overlap of the evidence base.

The descriptive distribution in Figure 3 complements the multilevel analysis by showing substantial within-family variability and extensive overlap among microbial targets. Very low MIC values occur in several bacteriocin families, indicating potentially strong activity for specific bacteriocin-target combinations, although the markedly unequal representation of families and targets limits direct potency comparisons. This heterogeneity is biologically plausible because susceptibility depends not only on bacteriocin family but also on strain characteristics, receptor availability, membrane composition, physiological state, peptide properties, and experimental conditions.

Differences in mechanisms of action further support this interpretation. Class IIa pediocin-like bacteriocins typically depend on receptor-mediated recognition, including components of the mannose phosphotransferase system, whereas lantibiotics such as nisin can interact with lipid II and combine inhibition of cell-wall biosynthesis with pore formation. Other peptides, including thiopeptides such as micrococcin, primarily interfere with translation. This mechanistic diversity is a major strength of bacteriocins because it provides opportunities to select molecules with complementary modes of action according to the biological characteristics of the target microorganism.

The broad nomenclature-based search strategy extended the evidence mapping beyond the most frequently investigated bacteriocins and included sakacins, pediocins, leucocins, curvacins/curvaticins, lacticin 3147, bavaricins, and carnobacteriocins. Representative translational examples include sakacin K in dry fermented sausages, pediocin AcH in sterile ground beef, sausage mixtures, and dairy systems, lacticin 3147 preparations in yogurt, cottage cheese, and other food systems, and purified leucocin A applied to wieners [23,24,25,26]. These studies demonstrate that the available evidence extends from intrinsic antimicrobial activity to practical testing in different food systems.

The food matrix is, however, a major determinant of antimicrobial performance and can generate substantial differences between MIC values measured in vitro and efficacy observed in situ. Sakacin P and nisin, for example, can rapidly adsorb to food proteins, while proteolytic degradation may reduce recoverable antimicrobial activity in untreated food matrices [27]. Fat content, protein binding, salt concentration, pH, oxidative state, protease activity, spatial distribution, and diffusion can all influence the effective concentration reaching microbial cells. Broth MIC values should therefore be regarded primarily as indicators of intrinsic susceptibility, whereas food challenge studies are essential for establishing technological efficacy and realistic application conditions.

For Gram-negative targets, the outer membrane represents an additional permeability barrier that can substantially limit apparent bacteriocin susceptibility. Sensitization strategies involving EDTA-mediated divalent-cation chelation, lactoferrin, organic acids, essential-oil components, heat treatment, freezing, high pressure, and other physical hurdles may increase bacteriocin access to the cytoplasmic membrane [8,28]. Such combinations are particularly relevant for food applications, where bacteriocins may function most effectively as components of integrated hurdle strategies. Nevertheless, effects observed in multi-component systems should be interpreted as combined-hurdle evidence rather than attributed to the bacteriocin alone.

Overall, bacteriocin family provides a useful framework for organizing the evidence base and identifying biologically relevant patterns, while the observation-level multilevel model allows these patterns to be evaluated without treating biologically distinct MIC observations as interchangeable replicates. Bacteriocin identity, microbial strain, assay conditions, and converted MIC values are transparently retained in Supplementary Tables S3, S9, and S21. Clinical or laboratory strains lacking explicit foodborne characterization are interpreted as antimicrobial-spectrum evidence rather than as direct proof of control of specific toxin-mediated foodborne diseases.

Taken together, the findings support a favorable outlook for the development of bacteriocins as targeted antimicrobial tools. The significant effects of bacteriocin family and target category, together with the substantial body of direct food-matrix evidence, provide a structured quantitative and biological basis for selecting promising bacteriocin-target combinations for further validation. Rather than identifying a universally superior bacteriocin, the evidence supports a targeted strategy in which bacteriocin identity, microbial strain, mechanism of action, and food-matrix conditions are considered jointly. Greater methodological standardization and well-controlled food challenge studies should facilitate the identification of bacteriocin-target-strain-matrix combinations with the greatest translational potential.

### 4.2. Translation to Animal-Derived Foods: Matrix Effects, Delivery and Hurdle Strategies

The mapping of food-matrix applications highlights a substantial degree of progress toward the practical use of bacteriocins. Among the 192 unique studies conducted in animal-derived or mixed matrices, 74 evaluated direct bacteriocin/BLIS addition, 67 combined-hurdle strategies, 33 producer/protective cultures, and 18 delivery systems. This distribution shows that research has explored multiple application routes, from assessing intrinsic efficacy within the matrix to combining bacteriocins with other preservation interventions, as well as the use of live cultures and controlled-release systems. In the dedicated 199-study dose-versus-MIC audit, five records allowed a direct within-study ratio or equivalence interpretation, whereas the remaining cases highlighted the need to interpret applied doses in relation to the specific characteristics of the matrix and delivery system. Overall, these findings emphasize the value of food challenge tests as a direct and biologically relevant measure of practical efficacy.

Interaction with the food matrix is a central determinant of bacteriocin performance. Protein and lipid binding, pH, salt content, protease activity, and diffusion can modulate the fraction of peptide that remains effectively available, whereas approaches such as surface application, encapsulation, coatings, and active films can enhance local concentration and persistence. Sakacin P and nisin, for example, can rapidly interact with food constituents [27], illustrating how real-world efficacy depends on the balance between molecular properties, matrix characteristics, and mode of delivery. This complexity makes food-based studies particularly valuable because they help identify conditions of use that more closely reflect practical applications.

Combined-hurdle strategies appear especially promising against Gram-negative bacteria. Interventions such as EDTA-mediated chelation, lactoferrin, organic acids, chitosan, essential-oil components, heat, freezing, high pressure, and other physical treatments can increase outer-membrane permeability and facilitate bacteriocin access to the cytoplasmic membrane [8,28]. Integrating bacteriocins with other hurdles therefore offers a practical opportunity to broaden antimicrobial activity and achieve synergistic effects under conditions compatible with product quality. Although co-treatments require appropriate experimental designs to distinguish the contribution of individual components, they also represent one of the major strengths of modern biopreservation strategies.

Producer cultures also represent an important application route, particularly when bacteriocin production occurs directly within the food matrix. In these cases, strain-level assessment is an essential step to ensure safety and reproducibility. Virulence determinants, antimicrobial-resistance genes, transferability of resistance, toxigenic potential, genome stability, and regulatory status should all be considered before food use. This is particularly relevant for enterococci, for which EFSA QPS assessment does not automatically provide a species-level presumption of safety [29]. Careful characterization can therefore help identify producer strains with favorable profiles in terms of both antimicrobial activity and safety.

The diversity of translational examples further confirms the breadth of application potential. In addition to enterocins and plantaricins, the literature includes sakacins, pediocins, leucocins, curvacins/curvaticins, lacticin 3147, bavaricins, and carnobacteriocins. Representative examples include sakacin K in dry fermented sausages, pediocin AcH in meat- and dairy-based model systems, lacticin 3147 preparations in dairy products, and purified leucocin A applied to wieners [23,24,25,26]. These studies demonstrate that bacteriocins can be integrated into different technological contexts through purified peptides, producer cultures, delivery systems, or combined-hurdle strategies.

Regulatory and technological readiness differs across bacteriocins, but the established experience with nisin demonstrates that industrial use of these compounds is feasible. For many other bacteriocins, progressive characterization of stability, manufacturing reproducibility, sensory compatibility, delivery, and safety is likely to support further practical development. In this context, the distinction between antimicrobial efficacy, regulatory safety, and technological feasibility should not be viewed as a barrier, but rather as the validation pathway required to transform promising candidates into usable interventions.

Overall, the available evidence supports bacteriocins as versatile and promising tools for matrix-specific biopreservation. Their value lies in the possibility of using them in a targeted manner, either alone or within hurdle-based strategies, by adapting the molecule, dose, and delivery system to the characteristics of the food product and the target microorganism. The breadth of studies already conducted in real food matrices therefore provides a strong foundation for developing increasingly effective, reproducible, and technologically compatible preservation strategies.

### 4.3. Emerging Antiparasitic Evidence: Therapeutic Models Are Not Food Biopreservation

The antiparasitic search identified direct in vitro and murine evidence for bacteriocin preparations against *Giardia lamblia* in Amer et al. [12], with the *L. acidophilus* P106 preparation showing greater activity than the *L. plantarum* P164 preparation at the tested doses. The search strategy also covered *Giardia*/*G. lamblia*/*G. intestinalis*/*G. duodenalis*, as well as *Taenia* and *Sarcocystis*, allowing parasite targets with no identified evidence to be documented explicitly.

*Trichinella* nevertheless represents the dominant parasite model, with nine publications identified. Much of this literature investigates live bacteriocinogenic or probiotic strains in murine models, often with a substantial focus on host immune responses. These effects should therefore be distinguished from direct activity of an isolated bacteriocin. The AP7121 study illustrates this point particularly clearly: the live *E. faecalis* CECT7121 strain showed antinematodic activity, whereas isolated AP7121 showed no larvicidal effect at the tested concentrations [14], demonstrating that the biological activity of a producer strain cannot automatically be attributed to its named bacteriocin.

The most direct peptide-level evidence is provided by the *Giardia* bacteriocin preparations [12] and by separately administered enterocin M and durancin-like preparations in experimental trichinellosis [15,16]. In contrast, the 2015 *L. plantarum* P164 study, earlier producer-strain investigations, and the 2025 *L. brevis*/*B. subtilis* study provide producer-strain or indirect probiotic evidence [17,18,19,20,21,22]. The 2025 *Cryptosporidium* preprint was retained only as sensitivity-tier evidence because the tested cell-free supernatants contained multiple components and the bacteriocin contribution was not isolated [13].

Importantly, none of the included antiparasitic studies evaluated parasite control directly in a food matrix. The available findings therefore provide a promising mechanistic basis for further investigation rather than direct evidence of food biopreservation efficacy. Future studies should identify and characterize the active peptide, confirm its production through genomic and peptidomic approaches, compare purified bacteriocins directly with their producer strains, adopt standardized parasite-stage endpoints, and establish safety. Testing the most promising candidates in relevant animal-derived food matrices would represent the key next step toward determining whether the observed antiparasitic activity can be translated into practical protective-culture or biopreservation strategies.

### 4.4. Limitations

Several limitations should be considered when interpreting this review. MIC methods varied across studies in growth medium, inoculum, incubation conditions, endpoint determination, target strain characterization, and bacteriocin preparation and purity. True observation-level sampling variances were generally unavailable, and multiple MIC observations from the same study often represented biologically distinct bacteriocin–strain combinations rather than technical replicates. For this reason, the primary analysis did not construct inverse-variance weights from within-study biological dispersion; instead, dependence was modeled through study, study–strain, and bacteriocin random intercepts. Although the additional study–strain and bacteriocin variance components were non-zero, they were small relative to the study-level component, and many bacteriocin families were sparsely and unevenly represented, limiting precision for underrepresented families. The target-specific study-level summaries are descriptive and should not be interpreted as pooled causal or potency estimates. Differences in strain, serovar/pathotype, assay protocol, and bacteriocin preparation therefore continue to constrain direct biological comparisons across studies. Food-application studies differ in matrix composition, dose expression, delivery mode, storage conditions, and the use of additional hurdles, meaning that broth MIC values cannot be directly converted into effective doses in food matrices. Producer-culture studies additionally require strain-specific safety and regulatory assessment. The antiparasitic evidence remains limited, mechanistically heterogeneous, and largely based on in vitro, ex vivo, or animal models rather than food-matrix challenge studies; it should therefore be regarded as preliminary and hypothesis-generating. In addition, no prospective review protocol was registered.

### 4.5. Future Quantitative Research Directions

Given the fragmented and inconsistent reporting of replicate-level MIC data across the available literature, future quantitative syntheses would benefit from more standardized reporting of replicate-level MIC values, MIC-specific uncertainty estimates, and key methodological variables, including bacteriocin preparation and purity, target strain, inoculum, growth medium, incubation conditions, and endpoint definition. In the present review, a conservative approach was adopted by avoiding the construction of sampling variances or inverse-variance weights not directly supported by the source data. If future primary studies provide replicate-level MIC values or valid MIC-specific uncertainty estimates together with sufficient replication, the multilevel framework used here could be further extended by incorporating additional methodological moderators and, where appropriate, cluster-robust variance estimation. Such developments could allow a clearer separation of study-, bacteriocin-, strain-, methodological-, and residual sources of variability.

## 5. Conclusions

In conclusion, this systematic review provides an integrated framework for the study and application of bacteriocins in food safety. A curated observation-level dataset was established to organize MIC evidence for bacteriocins tested against foodborne and food-relevant bacterial targets, while the available applications of bacteriocins in food matrices were systematically mapped and critically synthesized. The multilevel statistical analysis further strengthened this evidence base by accounting for study-, strain-, and bacteriocin-level dependence and by demonstrating significant overall effects of both bacteriocin family and target category. Together, these resources move the field beyond a simple collection of heterogeneous MIC values and provide a structured basis for identifying and comparing biologically relevant bacteriocin–target–strain–matrix combinations and for guiding their prioritization in future studies.

At the same time, this review organizes an emerging and still underexplored area of bacteriocin research: antiparasitic activity. Although the current evidence remains preliminary and is not yet sufficient to support direct food-biopreservation claims, the identification of activity in Giardia and Trichinella models highlights a promising direction for future investigation. Dedicated in vivo studies testing purified bacteriocin molecules, well-characterized producer strains, and their mechanisms of action will be particularly important to determine whether these effects can be translated into practical strategies for controlling parasitic hazards along the food chain. Overall, the combined quantitative, biological, and application-oriented framework developed here provides both a consolidated evidence base and a roadmap for more targeted, standardized, and translational bacteriocin research.

## Figures and Tables

**Table 1:** Target-level coverage for predefined target groups in the primary MIC tier (282 of 312 observations). The remaining 30 primary observations involve targets outside the predefined hazard panel. Counts refer to exact conventional MIC observations in directly convertible units; strain/serovar/pathotype details and individual MICs are provided in Supplementary Table S3.

Target	MIC Observations	Distinct Bacteriocins	Studies
*Staphylococcus aureus*	81	59	52
*Listeria monocytogenes*	124	50	34
*Escherichia coli*	35	29	29
*Salmonella* spp.	19	18	18
*Campylobacter* spp.	0	0	0
*Yersinia* spp.	0	0	0
*Clostridium perfringens*	4	4	2
*Bacillus cereus*	19	6	6
*Clostridium botulinum*	0	0	0

**Table 2:** Descriptive target-specific summaries of study-target mean log10 MIC values. Values are unweighted across study-target cells and are provided only to describe the distribution of study-level means; they are not treated as pooled effect sizes and are not used for inverse-variance inference.

Target	Study-Target Cells (k)	Unweighted Mean log10 MIC	Back-Transformed Mean, mg/mL	Range of Study-Target Mean log10 MIC	Interpretation
*Staphylococcus aureus*	9	−2.114	0.00768	−3.075 to −1.269	Descriptive only; no inverse-variance weighting
*Escherichia coli*	3	−2.258	0.00553	−3.404 to −1.325	Descriptive only; no inverse-variance weighting
*Listeria monocytogenes*	6	−2.025	0.00944	−2.682 to −1.522	Descriptive only; no inverse-variance weighting

**Table 3:** Final observation-level linear mixed-effects model results. The model used log10 MIC as the dependent variable, bacteriocin family and target category as fixed effects, and random intercepts for Study_ID, Study_Strain, and Bacteriocin. Model fit: REML deviance 495.3, log likelihood −247.6, AIC 539.3, BIC 621.6. Fixed effects were tested using Type III sums of squares with Satterthwaite degrees of freedom.

Result	Estimate/Statistic	SE/SD	df	95% CI/Variance	*p*
Global fixed effects (Type III tests; Satterthwaite degrees of freedom)					
Bacteriocin family	F = 3.568	—	11, 52.58	—	<0.001
Target category	F = 4.219	—	6, 115.42	—	<0.001
Model fit statistics					
REML deviance	495.3	—	—	—	—
Log likelihood/AIC/BIC	−247.6	—	—	AIC = 539.3; BIC = 621.6	—
Random-effects variance components					
Study_ID intercept	—	SD = 1.048	—	Variance = 1.098	—
Study_Strain intercept	—	SD = 0.124	—	Variance = 0.015	—
Bacteriocin intercept	—	SD = 0.123	—	Variance = 0.015	—
Residual	—	SD = 0.314	—	Variance = 0.099	—

**Table 4:** Direct animal-derived or mixed food-matrix applications classified by intervention type. Counts are unique studies; full article-level references, DOI links, doses and outcomes are provided in Supplementary Tables S5–S7.

Intervention Category	N	Typical Matrices/Implementation	Interpretation Relative to MIC
Direct bacteriocin/BLIS addition	74	Meat, milk/dairy/cheese, fish/seafood and mixed food models	Direct in-matrix challenge evidence, but dose depends on binding, diffusion, pH, salt and proteolysis; not assumed equivalent to broth MIC.
Combined hurdle/multi-component treatment	67	Refrigeration, acids, essential oils, heat, high pressure, cold plasma, chelators or other co-treatments	Observed efficacy cannot be assigned to the bacteriocin alone unless factorial controls isolate its contribution.
Producer/protective culture	33	Fermented meat, cheese/dairy, seafood and other challenge-test systems	Live-strain outcome may reflect bacteriocin plus other metabolites/competition; strain-specific safety assessment required.
Delivery system/active packaging or coating	18	Films, coatings, multilayers, encapsulation and surface delivery	Performance depends on release kinetics and surface loading; generally not numerically comparable with broth MIC.

**Table 5:** Antiparasitic evidence mapped by publication, parasite, intervention, evidence category and experimental model. Direct peptide-exposure evidence is distinguished from bacteriocinogenic/producer-strain and indirect probiotic/postbiotic evidence; peptide exposure does not by itself establish direct parasiticidal activity.

Ref.	Parasite	Intervention	Evidence Category	Model	Key Result/Interpretation
[12]	*Giardia lamblia*	Bacteriocins from *L. acidophilus* P106 and *L. plantarum* P164	Direct peptide exposure	In vitro + murine	P106: 58.3 ± 4.04% trophozoite reduction at 50 μg/mL; 81.63% reduction after five 50 μg/mouse doses. P164 weaker.
[13]	*Cryptosporidium parvum*	Mixed probiotics and cell-free supernatants	Sensitivity-tier postbiotic preprint	Murine	Mixed CFS gave the largest oocyst-shedding reduction; bacteriocin contribution is inseparable from enzymes/other metabolites.
[14]	*Trichinella spiralis*	*E. faecalis* CECT7121 and AP7121	Direct peptide exposure + producer strain	In vitro + murine	Live strain reduced larval viability/burden; isolated AP7121 showed no larvicidal effect at tested concentrations.
[15]	*Trichinella spiralis*	Enterocin M, durancin-like + producer enterococci	Direct peptide exposure + producer strain	Murine	Enterocins and strains stimulated phagocytosis/respiratory burst; mechanistic direct-peptide evidence.
[16]	*Trichinella spiralis*	Enterocin M, durancin-like + producer enterococci	Direct peptide exposure + producer strain	Murine + ex vivo/in vitro	Adult reductions 43.8% and 16.4% for enterocin M and durancin-like; larval reductions 39.6% and 15.0%, respectively.
[17]	*Trichinella spiralis*	*E. faecium* CCM8558; *E. durans* ED26E/7; *L. plantarum* 17L/1; *L. fermentum* CCM7421	Bacteriocinogenic/producer-strain (CCM7421 probiotic-only)	Murine	CCM8558 adult reduction 65% on d11; larval reductions 64%/49% on d25/d32; other strains also reduced larval burden.
[18]	*Trichinella spiralis*	Six probiotic strains including CCM8558 and ED26E/7	Bacteriocinogenic/probiotic strains	Murine + ex vivo/in vitro	CCM8558 larval reductions 74%/56%; CCM8558 and ED26E/7 reduced female fecundity by about 94%.
[19]	*Trichinella spiralis*	*E. faecium*/*E. durans*/*Lactobacillus* strain panel	Producer/probiotic-strain mechanistic evidence	Murine	Strain-dependent modulation of macrophage superoxide production; mechanistic host-defense endpoint.
[20]	*Trichinella spiralis*	CCM8558, ED26E/7, CCM7421, 17L/1	Producer/probiotic-strain mechanistic evidence	Murine	*L. fermentum* and *L. plantarum* restored CD4+ cells; all strains stimulated CD8+ responses. No isolated peptide tested.
[21]	*Trichinella spiralis*	*L. brevis* PQ214320; *B. subtilis* PQ198038	Indirect probiotic-strain sensitivity evidence	Murine	Strong parasite reductions reported, but no bacteriocin was isolated or established as the causal factor in the current experiment.
[22]	*Trichinella spiralis*	*L. plantarum* P164; *L. acidophilus* P110; *L. casei* ATCC 7469	Indirect/known-producer strain	Murine	P164 adult reductions 56.98–69.02%; larval reduction 87.92%. Current experiment used live probiotics, not isolated bacteriocin.

## Data Availability

All extracted MIC observations, food-matrix applications, antiparasitic evidence, search/deduplication audits, access/exclusion audit, observation-level quantitative analysis inputs, descriptive study-target summaries, and multilevel-analysis outputs are provided in the Supplementary Materials.

## Supplementary Materials

The following supporting information can be downloaded at: https://www.mdpi.com/article/10.3390/foods15203582/s1, PRISMA 2020 Checklist: Completed PRISMA 2020 checklist for this systematic review. Table S1: Search Strategies. Table S2: PRISMA Flow. Table S3: MIC Data. Table S4: Quality Appraisal. Table S5: Food Applications. Table S6: Dose vs MIC. Table S7: Food Reference Index. Table S8: Exclusion Coding. Table S9: Multilevel Input. Table S10: Target Coverage. Table S11: Family Target Map. Table S12: Antipara Evidence. Table S13: Antipara Quant. Table S14: Antipara Search. Table S15: Antipara ZeroEvidence. Table S16: Antipara Summary. Table S17: Evidence Verification. Table S18: Reference Inventory. Table S19: AbstractOnly Audit. Table S20: Access Exclusion Audit. Table S21: StudyTarget Descriptive. Table S22: Multilevel Results. Table S23: Sensitivity Audit.. References [12,13,14,15,16,17,18,19,20,21,22,23,26,30,31,32,33,34,35,36,37,38,39,40,41,42,43,44,45,46,47,48,49,50,51,52,53,54,55,56,57,58,59,60,61,62,63,64,65,66,67,68,69,70,71,72,73,74,75,76,77,78,79,80,81,82,83,84,85,86,87,88,89,90,91,92,93,94,95,96,97,98,99,100,101,102,103,104,105,106,107,108,109,110,111,112,113,114,115,116,117,118,119,120,121,122,123,124,125,126,127,128,129,130,131,132,133,134,135,136,137,138,139,140,141,142,143,144,145,146,147,148,149,150,151,152,153,154,155,156,157,158,159,160,161,162,163,164,165,166,167,168,169,170,171,172,173,174,175,176,177,178,179,180,181,182,183,184,185,186,187,188,189,190,191,192,193,194,195,196,197,198,199,200,201,202,203,204,205,206,207,208,209,210,211,212,213,214,215,216,217,218,219,220,221,222,223,224,225,226,227,228,229,230,231,232,233,234,235,236,237,238,239,240,241,242,243,244,245,246,247,248,249,250,251,252,253,254,255,256,257,258,259,260,261,262,263,264,265,266,267,268,269,270,271,272,273,274,275,276,277,278,279,280,281,282,283,284,285,286,287,288,289,290,291,292,293,294,295,296,297,298,299,300,301,302,303,304,305,306,307,308,309,310,311,312,313,314,315,316,317,318,319,320,321,322,323,324,325,326,327,328,329,330,331,332,333,334,335,336,337,338,339,340,341,342,343,344,345,346,347,348,349,350,351,352,353,354,355,356] are cited in the Supplementary Materials.
