# Supplementary material for: Bacteriocins for Safety of Animal-Derived Foods: Systematic Mapping, Multilevel MIC Analysis, Food-Matrix Applications, and Emerging Antiparasitic Evidence
Source: Foods. 2026 Oct 9;15(20):3582. doi: 10.3390/foods15203582 (PMC13703170; doi:10.3390/foods15203582)
Supplement: Supplementary file 1 [file foods-15-03582-s001.zip › PRISMA_2020_FINAL.pdf]

PRISMA 2020 Checklist

Bacteriocins for Safety of Animal-Derived Foods: Systematic Mapping, Multilevel MIC Analysis, Food-Matrix Applications, and Emerging Antiparasitic Evidence

Section and Topic	Item #	Checklist item	Location where item is reported
**TITLE**			
Title	1	Identify the report as a systematic review.	Yes - lines 2-5, Title. The report is explicitly identified as a systematic review; the quantitative component is described as a multilevel MIC analysis.
**ABSTRACT**			
Abstract	2	See the PRISMA 2020 for Abstracts checklist.	Partially reported - lines 15-39, Abstract. The abstract reports the review scope, databases searched, evidence-base sizes, the primary observation-level mixed-effects analysis, food-matrix findings, antiparasitic findings and main conclusions. Search dates, funding/registration information and the methodological/reporting appraisal are not stated in the abstract.
**INTRODUCTION**			
Rationale	3	Describe the rationale for the review in the context of existing knowledge.	Yes - lines 43-78, Introduction. The rationale is presented in the context of foodborne hazards, biopreservation, matrix dependence, antimicrobial heterogeneity and the limited antiparasitic evidence.
Objectives	4	Provide an explicit statement of the objective(s) or question(s) the review addresses.	Yes - lines 79-93, Introduction. Four explicit objectives are stated: systematic antibacterial evidence mapping; observation-level multilevel MIC analysis with descriptive study-target summaries; food-matrix application mapping; and emerging antiparasitic evidence mapping.
**METHODS**			
Eligibility criteria	5	Specify the inclusion and exclusion criteria for the review and how studies were grouped for the syntheses.	Yes - lines 94-138, 177-240, 243-273 and 274-291. Eligibility and grouping are defined separately for bacterial MIC evidence, the primary exact-MIC quantitative tier, descriptive study-target summaries, food-matrix applications, dose-versus-MIC comparability and antiparasitic evidence categories.
Information sources	6	Specify all databases, registers, websites, organisations, reference lists and other sources searched or consulted to identify studies. Specify the date when each source was last searched or consulted.	Yes - lines 143-176. PubMed, Scopus and Web of Science are identified; the original pathogen-specific searches were last run on 26 January 2026, the expanded Scopus search on 4 September 2026, and the expanded PubMed and Web of Science searches on 5 September 2026. Complete database-specific strategies are in Supplementary Tables S1 and S14.
Search strategy	7	Present the full search strategies for all databases, registers and websites, including any filters and limits used.	Yes - lines 143-176. The manuscript gives the core database syntax, expanded bacteriocin/pathogen/parasite terminology and search restrictions; complete database-specific search strings are provided in Supplementary Tables S1 and S14.
Selection process	8	Specify the methods used to decide whether a study met the inclusion criteria of the review, including how many reviewers screened each record and each report retrieved, whether they worked independently, and if applicable, details of automation tools used in the process.	Yes - lines 94-138 and 177-192. ChatGPT (OpenAI) is reported solely as a prioritization aid and not as an independent eligibility criterion. All deduplicated records underwent manual title/abstract assessment by two reviewers; full-text eligibility assessment and data extraction were performed independently by two reviewers, with disagreements resolved by consensus. Screening/access/exclusion details are in Supplementary Tables S2, S8 and S20.
Data collection process	9	Specify the methods used to collect data from reports, including how many reviewers collected data from each report, whether they worked independently, any processes for obtaining or confirming data from study investigators,	Yes - lines 109-138 and 193-240. Full-text eligibility assessment and data extraction were independently performed by two reviewers,

From: Page MJ, McKenzie JE, Bossuyt PM, Boutron I, Hoffmann TC, Mulrow CD, et al. The PRISMA 2020 statement: an updated guideline for reporting systematic reviews. BMJ 2021;372:n71. doi: 10.1136/bmj.n71. This work is licensed under CC BY 4.0. To view a copy of this license, visit https://creativecommons.org/licenses/by/4.0/

Section and Topic	Item #	Checklist item	Location where item is reported
		and if applicable, details of automation tools used in the process.	with disagreements resolved by consensus. Observation-level variables, quantitative evidence tiers and rules for unavailable full texts are specified; unreported information was not inferred.
Data items	10a	List and define all outcomes for which data were sought. Specify whether all results that were compatible with each outcome domain in each study were sought (e.g. for all measures, time points, analyses), and if not, the methods used to decide which results to collect.	Yes - lines 193-240, 243-273 and 274-291. Outcomes sought include exact conventional MIC observations, descriptive antibacterial study-target summaries, direct food-matrix application outcomes, dose-versus-MIC comparability, and categorized antiparasitic evidence.
	10b	List and define all other variables for which data were sought (e.g. participant and intervention characteristics, funding sources). Describe any assumptions made about any missing or unclear information.	Yes - lines 193-240, 243-273 and 274-291. Variables include title/DOI, bacteriocin identity and preparation/purity, target species and strain/serovar/pathotype when available, MIC and units, matrix, intervention type, antiparasitic evidence category and source information. Missing or unclear information was not inferred.
Study risk of bias assessment	11	Specify the methods used to assess risk of bias in the included studies, including details of the tool(s) used, how many reviewers assessed each study and whether they worked independently, and if applicable, details of automation tools used in the process.	Partially reported as a methodological/reporting appraisal - lines 255-265. An adapted predefined nine-domain appraisal was applied to the 94 studies contributing primary quantitative MIC observations and is explicitly not treated as a validated clinical risk-of-bias tool. Study-level results are in Supplementary Table S4. The number of reviewers performing this appraisal and whether they worked independently are not separately specified.
Effect measures	12	Specify for each outcome the effect measure(s) (e.g. risk ratio, mean difference) used in the synthesis or presentation of results.	Yes - lines 193-240. The primary quantitative outcome is individual log10 MIC (mg/mL). Bacteriocin-family and target-category effects are evaluated as global fixed effects in a one-stage linear mixed-effects model, and random-effects variance components are reported. Study-target mean log10 MIC values are presented only as unweighted descriptive summaries and are not used for inverse-variance inference.
Synthesis methods	13a	Describe the processes used to decide which studies were eligible for each synthesis (e.g. tabulating the study intervention characteristics and comparing against the planned groups for each synthesis (item #5)).	Yes - lines 193-240 define eligibility for the primary exact-MIC observation-level analysis and the descriptive study-target summaries; lines 243-273 define food-matrix and dose-versus-MIC categories; lines 274-291 define antiparasitic evidence categories.
	13b	Describe any methods required to prepare the data for presentation or synthesis, such as handling of missing summary statistics, or data conversions.	Yes - lines 193-240. MIC units are converted to mg/mL when directly valid, exact conventional values form the primary quantitative tier, and eligible MICs are log10-transformed. Censored/range values, arbitrary-unit endpoints, partially characterized preparations, mixtures, formulation-dependent endpoints and non-conventional values are reported transparently outside the primary inferential model or as sensitivity-tier evidence.
	13c	Describe any methods used to tabulate or visually display results of individual studies and syntheses.	Yes - lines 313-446 and 456-529. Results are displayed using a bacteriocin-family x target heatmap (Figure 2), descriptive study-target summaries (Table 2), an observation-level MIC distribution plot (Figure 3), multilevel model results and adjusted estimated marginal means (Table 3/Figure 4), and food-matrix/intervention summaries (Table 4/Figure 5).
	13d	Describe any methods used to synthesize results and provide a rationale for the choice(s). If meta-analysis was performed, describe the model(s), method(s) to identify the presence and extent of statistical heterogeneity, and software package(s) used.	Yes - lines 193-240. The primary synthesis is a one-stage observation-level linear mixed-effects model in JASP 0.98.1 with log10 MIC as the dependent variable, bacteriocin family and broad target category as fixed effects, and random intercepts for Study_ID, Study_Strain and bacteriocin identity. The model is fitted by REML; global fixed effects use Type III tests with Satterthwaite degrees of freedom. Study-target summaries are descriptive only and are not used for inverse-variance inference.
	13e	Describe any methods used to explore possible causes of heterogeneity among study results (e.g. subgroup	Partially reported - lines 193-240. Bacteriocin family and target category are modeled as fixed effects, while study, study-strain and

From: Page MJ, McKenzie JE, Bossuyt PM, Boutron I, Hoffmann TC, Mulrow CD, et al. The PRISMA 2020 statement: an updated guideline for reporting systematic reviews. BMJ 2021;372:n71. doi: 10.1136/bmj.n71. This work is licensed under CC BY 4.0. To view a copy of this license, visit https://creativecommons.org/licenses/by/4.0/

Section and Topic	Item #	Checklist item	Location where item is reported
		analysis, meta-regression).	bacteriocin identity are modeled as random intercepts to separate major sources of variability. Additional methodological moderators were not modeled systematically because reporting was incomplete/inconsistent; this is discussed as a future extension in Section 4.5 (lines 731-747).
	13f	Describe any sensitivity analyses conducted to assess robustness of the synthesized results.	No separate inferential sensitivity analysis is reported. Non-primary and formulation-dependent evidence is documented outside the primary exact-MIC tier, and the sensitivity evidence/analysis-boundary audit is provided in Supplementary Table S23.
Reporting bias assessment	14	Describe any methods used to assess risk of bias due to missing results in a synthesis (arising from reporting biases).	Not performed. The manuscript does not describe a formal assessment of reporting bias or risk of bias due to missing results.
Certainty assessment	15	Describe any methods used to assess certainty (or confidence) in the body of evidence for an outcome.	Not performed. No formal certainty-of-evidence framework or grading method is described.
**RESULTS**			
Study selection	16a	Describe the results of the search and selection process, from the number of records identified in the search to the number of studies included in the review, ideally using a flow diagram.	Yes - lines 292-312 and Figure 1. The review reports 25,499 source rows, 12,700 unique bibliographic records after deduplication, 1,591 unique records proceeding to retrieval/eligibility assessment, and 342 unique included publications across the reconciled evidence sets.
	16b	Cite studies that might appear to meet the inclusion criteria, but which were excluded, and explain why they were excluded.	Partially reported - lines 177-192 and 292-312 describe the access/exclusion process and refer to Supplementary Tables S2, S8 and S20. Exclusion coding is supplied, but an individual citation list of reports that appeared eligible and were excluded at full-text assessment is not provided.
Study characteristics	17	Cite each included study and present its characteristics.	Yes - lines 313-529 summarize characteristics of the antibacterial, food-matrix and antiparasitic evidence. Individual MIC observations are in Supplementary Table S3; primary multilevel inputs are in S9; food applications are in S5-S7; antiparasitic evidence is in S12-S16; descriptive study-target summaries are in S21; and the reconciled reference inventory is in S18.
Risk of bias in studies	18	Present assessments of risk of bias for each included study.	Partially reported as a methodological/reporting appraisal - lines 447-455. Study-level domain results for the 94 primary MIC studies are provided in Supplementary Table S4. No analogous validated risk-of-bias instrument is applied to the heterogeneous food-matrix or antiparasitic evidence streams.
Results of individual studies	19	For all outcomes, present, for each study: (a) summary statistics for each group (where appropriate) and (b) an effect estimate and its precision (e.g. confidence/credible interval), ideally using structured tables or plots.	Yes, as applicable - lines 313-446 and 488-529. Individual MIC observations and target/strain labels are reported in Supplementary Tables S3 and S9; descriptive study-target values are reported in Table 2 and S21; adjusted multilevel results are in Table 3/Figure 4 and S22. Food-matrix study details are in S5, and antiparasitic study-level evidence is in S12-S16.
Results of syntheses	20a	For each synthesis, briefly summarise the characteristics and risk of bias among contributing studies.	Yes - lines 364-455 summarize the descriptive quantitative evidence, the primary multilevel synthesis and methodological/reporting appraisal. Study-level appraisal is in Supplementary Table S4; primary multilevel inputs/results are in S9 and S22; descriptive study-target summaries are in S21.
	20b	Present results of all statistical syntheses conducted. If meta-analysis was done, present for each the summary estimate and its precision (e.g. confidence/credible interval) and measures of statistical heterogeneity. If comparing groups, describe the direction of the effect.	Yes - lines 406-446 and Table 3/Figure 4. The statistical synthesis reports significant global effects of bacteriocin family ($F(11, 52.58)=3.568$, $p<0.001$) and target category ($F(6, 115.42)=4.219$, $p<0.001$), together with Study_ID, Study_Strain, bacteriocin and residual variance components. The analysis is observation-level; target-specific inverse-variance pooled estimates are not used for inferential conclusions.

From: Page MJ, McKenzie JE, Bossuyt PM, Boutron I, Hoffmann TC, Mulrow CD, et al. The PRISMA 2020 statement: an updated guideline for reporting systematic reviews. BMJ 2021;372:n71. doi: 10.1136/bmj.n71. This work is licensed under CC BY 4.0. To view a copy of this license, visit https://creativecommons.org/licenses/by/4.0/

Section and Topic	Item #	Checklist item	Location where item is reported
	20c	Present results of all investigations of possible causes of heterogeneity among study results.	Partially reported - lines 406-446 report fixed effects for bacteriocin family and target category and variance decomposition across study, study-strain, bacteriocin and residual components. No dedicated analysis of additional methodological moderators is performed because these variables were not reported consistently enough across studies; this is addressed in Section 4.5.
	20d	Present results of all sensitivity analyses conducted to assess the robustness of the synthesized results.	No separate inferential sensitivity analysis is reported. Sensitivity-tier/non-primary evidence is documented in Supplementary Table S23 and is not combined with the primary exact-MIC tier when methodological equivalence is not established.
Reporting biases	21	Present assessments of risk of bias due to missing results (arising from reporting biases) for each synthesis assessed.	Not applicable/not performed - no formal assessment of reporting bias or missing-result bias is reported for the syntheses.
Certainty of evidence	22	Present assessments of certainty (or confidence) in the body of evidence for each outcome assessed.	Not applicable/not performed - no formal certainty-of-evidence assessment or rating is reported.
**DISCUSSION**			
Discussion	23a	Provide a general interpretation of the results in the context of other evidence.	Yes - lines 530-706, Discussion Sections 4.1-4.3. Results are interpreted in relation to biological mechanisms, study/strain heterogeneity, food-matrix translation, producer-strain safety and the emerging antiparasitic evidence.
	23b	Discuss any limitations of the evidence included in the review.	Yes - lines 707-730, Section 4.4 'Limitations'. Limitations include MIC-method heterogeneity, unavailable observation-level sampling variances, sparse/uneven representation of some bacteriocin families, strain/protocol variation, matrix/dose heterogeneity, producer-strain safety considerations, limited antiparasitic evidence and absence of prospective protocol registration.
	23c	Discuss any limitations of the review processes used.	Partially reported - lines 94-138 transparently describe the differential full-text retrieval strategy for low-priority records, while lines 707-730 note the absence of prospective protocol registration. The differential retrieval strategy is not explicitly revisited as a review-process limitation in Section 4.4.
	23d	Discuss implications of the results for practice, policy, and future research.	Yes - lines 575-706 and 731-747. The manuscript discusses food-specific challenge testing, hurdle design, producer-strain safety/regulatory assessment, antiparasitic validation, reporting standardization, replicate-level MIC uncertainty and potential future extensions of the multilevel framework.
**OTHER INFORMATION**			
Registration and protocol	24a	Provide registration information for the review, including register name and registration number, or state that the review was not registered.	Yes - lines 94-95 and 729-730 state that no prospective review protocol was registered.
	24b	Indicate where the review protocol can be accessed, or state that a protocol was not prepared.	Not fully reported. Lines 94-95 and 729-730 state that no prospective review protocol was registered, but the manuscript does not explicitly state whether a separate protocol was prepared or where it could be accessed.
	24c	Describe and explain any amendments to information provided at registration or in the protocol.	Not applicable on the basis of the reported information because no review registration is reported; protocol amendments are not described.
Support	25	Describe sources of financial or non-financial support for the review, and the role of the funders or sponsors in the review.	Yes - Funding statement, page 23. Magna Graecia University of Catanzaro and the University of Turin 'Grant for Internationalization 2025' are identified as funding sources; the role of the funders/sponsors in the review is not separately stated.
Competing interests	26	Declare any competing interests of review authors.	Yes - Conflicts of Interest statement, page 23. The authors declare no conflicts of interest.
Availability of data,	27	Report which of the following are publicly available and where they can be found: template data collection forms;	Partially reported - Supplementary Materials and Data Availability

From: Page MJ, McKenzie JE, Bossuyt PM, Boutron I, Hoffmann TC, Mulrow CD, et al. The PRISMA 2020 statement: an updated guideline for reporting systematic reviews. BMJ 2021;372:n71. doi: 10.1136/bmj.n71. This work is licensed under CC BY 4.0. To view a copy of this license, visit https://creativecommons.org/licenses/by/4.0/

PRISMA

PRISMA 2020 Checklist

Section and Topic	Item #	Checklist item	Location where item is reported
code and other materials		data extracted from included studies; data used for all analyses; analytic code; any other materials used in the review.	Statement, pages 22-23. Supplementary Tables S1-S23 provide extracted MIC data, food-matrix and antiparasitic evidence, search/access/exclusion audits, primary observation-level analysis inputs, descriptive study-target summaries, multilevel-analysis outputs and sensitivity-tier/audit material. Analytic code is not identified as a separately available item in the manuscript.

From: Page MJ, McKenzie JE, Bossuyt PM, Boutron I, Hoffmann TC, Mulrow CD, et al. The PRISMA 2020 statement: an updated guideline for reporting systematic reviews. BMJ 2021;372:n71. doi: 10.1136/bmj.n71. This work is licensed under CC BY 4.0. To view a copy of this license, visit https://creativecommons.org/licenses/by/4.0/
